# A Dispensatory, or Commentary on the Pharmacopœias of Great Britain; Comprising the Natural History, Description, Chemistry, Pharmacy, Actions, Uses, and Doses of the Articles of the Materia Medica

**Published:** 1842-04

**Authors:** 


Art. XII.—*A Dispensatory, or Commentary on the Pharmacopœias of Great Britain; comprising the Natural History, Description, Chemistry, Pharmacy, Actions, Uses, and Doses of the Articles of the Materia Medica.* By Robert Christison, m.d. f.r.s.e., Professor of Materia Medica in the University of Edinburgh.—*Edinburgh*, 1842. 8vo, pp. 978.

We have recently entered so largely on the subject of Materia Medica and Pharmacy, that we must dismiss, with a mere notice, the valuable volume now before us. We cannot do so, however, without expressing our unqualified praise of its plan and execution. Its plan is considerably different from that of the late Dr. Duncan's Edinburgh Dispensatory, and Dr. Thomson's London Dispensatory, and is a decided improvement on them. "Instead of the subject being divided into several parts, and each topic treated of under several distinct heads, a method which renders consultation troublesome, study perplexing, and frequent repetitions unavoidable,—it has been thought preferable to exhaust every special topic under a single head;"—in a word, the work is strictly alphabetical, and all the preparations of each article is given under the name of this article. In the natural and commercial history of the different drugs we have all the information that is requisite without any superfluous or irrelevant details; and under the head of "Actions and Uses," we have concise yet precise descriptions of the physiological effects and the therapeutic properties of the different remedies. In this department it is easy to recognize the results of the judgment and experience of the practised physician, not the bald repetitions of the mere compiler. The only work that can at all compete in point of excellence with this is Dr. Pereira's most elaborate Materia Medica; and for the mere practitioner, Dr. Christison's deserves the preference, on account of its greater conciseness and readier accessibility in consultation. We accordingly earnestly recommend Dr. Christison's Dispensatory to all our readers as an indispensable companion, not in the Study only, but in the "Surgery" also. We ought to add, that the volume, while "got up" in the very best style, has the great and too-rare merit of being, for its size, remarkably cheap.

